# Key Grazing Behaviours of Beef Cattle Identify Specific Genotypes of the Glutamate Metabotropic Receptor 5 Gene (*GRM5*)

**DOI:** 10.1007/s10519-023-10169-4

**Published:** 2024-01-16

**Authors:** Cristian Anibal Moreno García, Susana Beatríz Perelman, Robyn Dynes, Thomas M. R. Maxwell, Huitong Zhou, Jonathan Hickford

**Affiliations:** 1 Department of Agricultural Sciences, Faculty of Agriculture and Life Sciences, Lincoln University, Lincoln, Canterbury, New Zealand; 2 Departamento de Métodos Cuantitativos y Sistemas de Informacíon, Institute for Agricultural Plant Physiology and Ecology IFEVA CONICET, Universidad de Buenos Aires, CABA, Buenos Aires, Argentina; 3 Lincoln Research Centre, AgResearch Limited, Lincoln, Canterbury, New Zealand

**Keywords:** Animal personality, Behavioural genetics, Global positioning system tracking (GPS-tracking), Grazing personalities, Steep and rugged terrain, Quadratic discriminant analysis

## Abstract

Genotype-phenotype associations between the bovine genome and grazing behaviours measured over time and across contexts have been reported in the past decade, with these suggesting the potential for genetic control over grazing personalities in beef cattle. From the large array of metrics used to describe grazing personality behaviours (GP-behaviours), it is still unclear which ones are linked to specific genes. Our prior observational study has reported associations and trends towards associations between genotypes of the glutamate metabotropic receptor 5 gene (*GRM5*) and four GP-behaviours, yet the unbalanced representation of *GRM5* genotypes occurring in observational studies may have limited the ability to detect associations. Here, we applied a subsampling technique to create a genotypically-balanced dataset in a *quasi*-manipulative experiment with free ranging cows grazing in steep and rugged terrain of New Zealand’s South Island. Using quadratic discriminant analysis, two combinations of eleven GP-behaviours (and a total of fifteen behaviours) were selected to build an exploration model and an elevation model, respectively. Both models achieved ∼ 86% accuracy in correctly discriminating cows’ *GRM5* genotypes with the training dataset, and the exploration model achieved 85% correct genotype prediction of cows from a testing dataset. Our study suggests a potential pleiotropic effect, with *GRM5* controlling multiple grazing behaviours, and with implications for the grazing of steep and rugged grasslands. The study highlights the importance of grazing behavioural genetics in cattle and the potential use of *GRM5* markers to select individuals with desired grazing personalities and built herds that collectively utilize steep and rugged rangelands sustainably.

## Introduction

Grazing personalities of foraging animals were defined as ‘suites of traits of different nature (*e.g.*, behavioural, cognitive, physiological, and morphological), which are correlated and often concatenated, to result in specific grazing patterns displayed consistently across contexts and over time’ (Moreno García et al. 2020). In this context, the consistent expression of distinctive grazing personalities may in part be underpinned by specific grazing genes (Moreno García et al. 2020). The social and biophysical environments as well as the animal’s experiences and emotional states are likely to affect grazing behaviours at the individual and collective level.

The social and biophysical environments are strong drivers of grazing behaviours in herbivores (Raynor et al. 2021; Senft et al. 1987; Zhao and Jurdak 2016), which are further shaped by the cognitive condition of the animals, yet behavioural genes and their expression are also intrinsic determinants of behaviours that are passed inter-generationally as revealed in meta-analyses of animal personality in wild and domestic animal populations. For example, van Oers and Sinn (2013) reported animal personality trait heritability ranging from 0.24 in domestic populations, to 0.36 in wild populations after a meta-analysis of 75 studies of animal personality. Similarly, Dochtermann et al. (2015) targeted publications on animal personality with estimates of repeatability and heritability of animal behaviours. These authors concluded that despite the often moderate to low heritability of behaviour, repeated behaviours pertaining to animal personalities had much higher heritability with an estimated 52% of its variation explained by genetic variation. While grazing behaviours in cattle have been attributed to animal personality (Neave et al. 2018), its genetic basis seems to be poorly understood.

Howery et al. (1998) conducted horse-back observations of the habitat use of free-ranging cattle herds for four summer seasons in a grazing allotment in the Sawtooth National Forest (Idaho), USA. After observing the habitat use of dams, foster-dams and young offspring, the authors realized that different groups of cattle consistently prefer certain habitats over others. The authors recommended the culling of animals displaying ‘undesirable habitat use characteristics’, as a mean of improving the grazing distribution of cattle herds. Twenty years later, it was still unclear as to whether grazing behavioural differences observed among individual cattle were attributable to learned or inherited behaviours, or to a combination of both (Howery and Bailey 2018). However, the identification of genetic effects on grazing behavioural differences could be a cost-effective tool to shape the collective grazing behaviour of cattle herds through selection, and potentially contribute to improving cattle distribution in grazing lands.

Studies by Bailey et al. (2015) and Pierce et al. (2020) pioneered the use of whole genome screening of free-ranging cattle to attempt to identify genetic regions associated with terrain-use indexes. These indexes were derived from key grazing behaviours, such as a cow’s movement relative to *in-situ* elevation, slope, and distance to water sources. While these studies reported promising genetic associations and suggested potential quantitative trait loci (QTL) and candidate genes, the sample size in the Bailey et al. (2015) study was small (*n* = 87) suggesting the need for a larger investigation. Pierce et al. (2020) had a larger sample size (*n*= 321), but their results were not consistent with Bailey et al. (2015), and they reported only weak associations, possibly because of still having a relatively small sample size, and because of the heterogeneity of their grazing data.

Moreno García et al. (2020) targeted the glutamate metabotropic receptor 5 gene (*GRM5*), studying variation in the exon 5 region in over 300 cows (*n* = 303). They reported genetic associations between genotypes and the grazing behaviours of home range and movement tortuosity, with a trend towards association with elevation range and horizontal distance travelled. These findings, together with the earlier study of Bailey et al. (2015) and the reported associations of *GRM5* expression variation with activity levels and exploratory behaviours in animal models (Bakker and Oostra 2003; Jew et al. 2013; Wu et al. 2020), support the relevance of *GRM5* as a predictor of grazing personality behaviours in beef cattle.

Moreno García et al. (2020) analyses were performed in an observational study applying a mensurative approach and without control over the proportion of *GRM5* genotypes in the sampled cattle. This forced the authors to exclude from analysis a rare genotype present in just 1% of the cattle investigated and apply their modelling to an unbalanced dataset of five *GRM5* genotypes, where two genotypes accounted for 71% of the sampled cows.

Haixiang et al. (2017) describe the problems encountered by classification algorithms when dealing with unbalanced datasets, and among other solutions discuss ‘dataset under sampling’ (*i.e.*, randomly discarding cases of the majority classes) to obtain better balanced datasets that equally represent all classes under investigation. Such strategies leading to improved datasets interspersion were much earlier proposed by Gosset (1938) and Cox (1958), as more balanced datasets might reveal hitherto hidden differences that would otherwise be undetected. Accordingly, in this study, a *quasi*-manipulative experiment design was set by under-sampling to investigate bovine *GRM5* genotypes and grazing behaviours in beef cattle.

On the basis that grazing genes and their expression precede the development of individual and collective grazing personalities (Moreno García et al. 2020), the following study hypothesized that a combination of consistent grazing behaviours might reveal specific *GRM5* genotypes in beef cattle under free-range management. It was further hypothesized that genetic variation of *GRM5* would result in differences among cows for simple grazing personality behaviours. The aims were therefore to identify a combination of grazing behaviours that assist in discriminating *GRM5* genotypes in beef cattle, and to assess the behavioural differences that might exist between different *GRM5* genotypes. A further aim was to ascertain if temporal consistency is needed for selecting grazing behaviours that assist in the distinction of *GRM5* genotypes.

## Materials and Methods

The Lincoln University Animal Ethics Committee approved all procedures involving animal handling and sampling (AEC 201816, AEC 201816 extension and AEC 202002).

The investigation was conducted using a selected subsample of the cattle described in Moreno García et al. (2020), to create a *quasi*-manipulative experiment (Hurlbert 1984) where *GRM5* genotype was the treatment, farm was a block effect and individual cows of four and 5 years of age (*i.e.*, cow age class 2 in Moreno García et al. 2020) were the experimental unit. We combined the under-resampling method (the random discard of samples from the major classes) and the exclusion of possible outlier individuals (detected with the ‘aq.plot()’ function of the R package ‘mvoutlier’, Filzmoser et al. 2005; Filzmoser and Gschwandtner 2021) to build a balanced dataset. This was expected to improve the accuracy of discriminant models compared to using unbalanced data, which can compromise the performance of classification algorithms (Haixiang et al. 2017).

Two separate datasets were selected, a fully balanced training dataset and a testing dataset for validation. The training dataset included four cows for each *GRM5* genotype present at greater than a 5% threshold (*i.e.*, *AB*, *AC*, *BB*, *BC*, and *CC*) from each of the four farms (*n* = 80 cows in total) and thus evenly represented the five common *GRM5* genotypes with 16 cows per genotype. The testing dataset included the same five *GRM5* genotypes, but with a less balanced representation (*i.e.*, *AB*, *AC*, *BB*, *BC*, and *CC*; with *n* = 7, 16, 13, 16, and 16 cows respectively). These cows were still present on all four farms (*n* = 68).

The training dataset was purposely balanced to evenly represent the *GRM5* genetic variation as well as the four farms involved in the original study. However, in the testing dataset, there was unbalanced representation of the five genotypes on all four farms, with fewer cows that were *AB* and *BB*, than were *AC*, *BC*, and *CC* (*i.e.*, 11%, 23%, 19%, 23% and 23%, respectively).

Age-based variation in the data was minimized by selecting only cows of 4–5 years of age, which were expected to display the ‘stable grazing behaviours’ of mature animals described by Moreno García et al. (2020).

### Study Sites and Cattle

For a detailed description of the methods, refer to Moreno García et al. (2020). Briefly, the study involved four private farms located in the steep and rugged hill country terrain of Canterbury, New Zealand. The cows studied were randomly selected from existing commercial herds. The location of the grazing cows was at elevations ranging between 200 and 1000 metres (m) above sea level in relatively large and undeveloped paddocks of an average size of 34.5 hectares (ha). All the paddocks had at least one water supply (natural springs and streams were sometimes present).

The cows were tracked with home-made GPS units in tracking collars (modified i-gotU GT-600 loggers; Mobile Action) deployed over winter months (April–August) of 2019 and 2020 seasons. From each cow deployment, a trajectory including free-range grazing was created with the ‘adehabitatLT’ R package (Calenge 2006). Any GPS outliers were excluded based on turning angles and the speed of consecutive geolocations (Guo et al. 2009) and trajectory parameters were recalculated. Geolocations were annotated for elevation, slope and aspect using the ‘raster’ R package (Hijmans 2021) and derived rasters (3D Analyst toolbox; ArcMapTM, ESRI 2020) from digital elevation models of New Zealand (16 m × 16 m spatial resolution from the shuttle radar topography mission, Land Information New Zealand, LINZ_DATA_SERVICE 2022).

### Grazing Behaviours

The trajectories of the cows were used to calculate variables related to cattle grazing patterns and grazing distribution in free-range systems as potential descriptors of grazing personality behaviours (Table 1). The broad array of 35 variables were chosen based on their use in previous studies (Bailey and Provenza 2008; Bailey et al. 2006; Browning et al. 2018; Gillen et al. 1984; Senft 1989; Senft et al. 1983). Over the 15 consecutive days of GPS-based monitoring, the individual cow measurements were aggregated into daily mean values and coefficients of variation (CV) calculated using the ‘summary()’ function of ‘dplyr’ R package (Wickham et al. 2021). Variables that were abnormally distributed were excluded from further analysis. Table1 presents a summary of the calculations and data transformation used.

**Table 1:** List of grazing personality behaviours with abbreviations, units, data transformations and description of calculations

Grazing personality behaviors	Abbreviations	Units	Data transformation	Description
Daily horizontal distance travelled	dist_ho	m/d	Square root	Distance calculated as the sum of distances between consecutive GPS^a^ data points per day using two dimensions (*i.e.*, Easting and Northing) of the UTM^b^ projection
Daily vertical distance travelled	dist_ve	m/d	Log	Distance calculated as the sum of the absolute difference in elevation (*i.e.*, dimension z) between consecutive GPS data points per day using a DEM^c^
Daily elevation range	ele_range	m		Range of elevation computed as the difference between the daily maximum and minimum elevation
Daily elevation gain	ele_gain	m/d	Square root	Sum of positive changes of elevation between consecutive GPS data points as depicted from a DEM
Daily mean elevation centred per farm	ele_mean_farm	m		For any given cow of a farm, the mean elevation across GPS data points per day as depicted from a DEM minus the mean elevation for that farm calculated across all days and cows included in the analysis
Daily elevation 85th quantile centred per farm	ele85_farm	m		For any given cow of a farm, the 85th quantile of elevation across GPS data points per day as depicted from a DEM minus the mean elevation for that farm calculated across all days and cows included in the analysis
Daily elevation 15th quantile centred per farm	ele15_farm	m		For any given cow of a farm, the 15th quantile of elevation across GPS data points per day as depicted from a DEM minus the mean elevation for that farm calculated across all days and cows included in the analysis
Daily slope mean	slope_mean	°	Square root	Mean slope across GPS data points per day as depicted from a DEM
Daily slope maximum	slope_max	°	Cube root	Maximum slope registered in any given day across GPS data points as depicted from a DEM
Daily slope 85th quantile	slope85	°	Cube root	85th quantile of the slope across GPS data points per day as depicted from a DEM
Daily slope 15th quantile	slope15	°	Cube root	15th quantile of the slope across GPS data points per day as depicted from a DEM
Daily home range	hr_mcp	ha/d	Log	Explored area estimated by calculating the minimum convex polygon depicted from all GPS data points per day using the R package ’adehabitatHR’
Daily movement tortuosity	sp_tortuosity	m/ha	Log	Movement tortuosity using the spatial search pattern estimated as the ratio between daily horizontal distance and daily home range
Adjusted daily horizontal distance travelled	adj_dist_ho	m/d		In any given day, the cow’s horizontal distance minus the minimum horizontal distance recorded in the herd plus 3500 (*i.e.*, roughly the mean daily horizontal distance for all cows and days)
Adjusted daily elevation mean	adj_ele_mean	m	Square	In any given day, the cow’s mean elevation minus the minimum elevation recorded for the same day in the herd plus 350 (*i.e.*, roughly the mean daily elevation for all cows and days)
Relative elevation range	rel_ele_range	0–1 scale	Cube root	In any given day, the ratio between the cows’ elevation range and the elevation range of the herd
Relative elevation mean	rel_ele_mean	0–1 scale	Cube root	In any given day, ratio between the cows’ mean elevation minus the minimum elevation of the herd and, the elevation range of the herd
Relative slope range	rel_slope_range	0–1 scale	Cube root	In any given day, ratio between the cows’ slope range (*i.e.*, maximum minus minimum slope) and the slope range of the herd
CV^d^ of daily horizontal distance travelled	dist_ho_cv	0–1 scale		Coefficient of variation of dist_ho
CV of daily vertical distance travelled	dist_ve_cv	0–1 scale	Log	Coefficient of variation of dist_ve
CV of daily elevation range	ele_range_cv	0–1 scale	Log	Coefficient of variation of ele_range
CV of daily elevation gain	ele_gain_cv	0–1 scale	Log	Coefficient of variation of ele_gain
CV of daily elevation 85th quantile centred per farm	ele85_farm_cv	0–1 scale	Log	Coefficient of variation of ele85_farm
CV of daily elevation 15th quantile centred per farm	ele15_farm_cv	0–1 scale	Log	Coefficient of variation of ele15_farm
CV of daily slope mean	slope_mean_cv	0–1 scale	Log	Coefficient of variation of slope_mean
CV of daily slope maximum	slope_max_cv	0–1 scale	Log	Coefficient of variation of slope_max
CV of daily slope 85th quantile	slope85_cv	0–1 scale	Log	Coefficient of variation of slope85
CV of daily slope 15th quantile	slope15_cv	0–1 scale	Log	Coefficient of variation of slope15
CV of daily home range	hr_mcp_cv	0–1 scale	Log	Coefficient of variation of hr_mcp
CV of daily movement tortuosity	sp_tortuosity_cv	0–1 scale	Log	Coefficient of variation of sp_tortuosity
CV of adjusted daily horizontal distance travelled	adj_dist_ho_cv	0–1 scale	Log	Coefficient of variation of adj_dist_ho
CV of adjusted daily elevation mean	adj_ele_mean_cv	0–1 scale	Cube root	Coefficient of variation of adj_ele_mean
CV of relative elevation range	rel_ele_range_cv	0–1 scale	Log	Coefficient of variation of rel_ele_range
CV of relative elevation mean	rel_ele_mean_cv	0–1 scale	Cube root	Coefficient of variation of rel_ele_mean
CV of relative slope range	rel_slope_range_cv	0–1 scale	Log	Coefficient of variation of rel_slope_range

^a^GPS: global positioning system fixes recorded with i-gotU GT-600, Mobile Action

^b^UTM: universal transverse mercator

^c^DEM: digital elevation model with a 16 m × 16 m spatial resolution

^d^CV: coefficient of variation

The analysis included days with more than a 75% fix rate for a frequency set at 5 minutes (*i.e.*, at least 216 out of a maximum of 288 data points per day). Any days that included collar deployment, deliberate herding and stock movement, and general animal handling were excluded; so, the data only represented periods of free-range grazing for the cows. The grazing days were recorded in hill and high-country grasslands, which were labelled as such when the median daily slope of the space grazed by the herd was greater than 8 degrees (°; *i.e.*, rolling, or steeper slope classes in New Zealand grasslands, Newsome et al. 2008). Finally, only cow deployments with seven or more days of behavioural data were used for analysis, as this was deemed sufficient to represent consistent behaviour.

### Statistical Analysis

Statistical analyses were conducted with R (R-Core-Team 2020). For data wrangling, several functions of the following R packages were used, including ‘Reshape’ (Wickham 2007), ‘dplyr’ (Wickham et al. 2021), and ‘data.table’ (Dowle and Srinivasan 2021). Skewness, kurtosis, and the normality of grazing behaviours per *GRM5* genotype were evaluated with histograms and with Q-Q plots. When needed, data transformations were applied to better-fit raw values into normal distributions. The Shapiro-Wilk test of normality was performed using the ‘stat.desc()’ function from the ‘pastecs’ R package (Grosjean and Ibanez 2018).

Linear discriminant analyses (LDA) were performed with the R packages ‘MASS’ (Venables and Ripley 2002) and ‘DiscriMiner’ (Sanchez 2013) in a backward stepwise iteration that started with all grazing behaviours. Initially grazing behaviours were selected based on pooled discriminant scores and on the discriminant accuracy rate achieved by each model. Variables were further selected to avoid multi-collinearity with the variance inflation factors (VIF) threshold of < 10 calculated with the R package ‘car’ (Fox and Weisberg 2018). Homogeneity of covariance was assessed with Box’s M-test ‘heplots::boxM()’ R function (Friendly 2010) and a final model applying quadratic discriminant analysis (QDA) was built with ‘MASS’ and the ‘DiscriMiner’ packages.

Multivariate analyses of variance (MANOVA) were performed with the R package ‘PERMANOVA’ (Vicente-Gonzalez and Vicente-Villardo 2021) to graphically identify variable redundancies and importance in the MANOVA map, and to assess the amount of total variation explained by the selected combination of variables. Test of multivariate normality per genotype was performed using the ‘byf.shapiro()’ function from the R package ‘RVAideMemoire’ (Hervé 2022).

Two-way analyses of variance (ANOVA) were performed with *GRM5* genotypes or with *GRM5* variant presence/absence as main effects, and with farm id as covariate (block effect) using the ‘aov()’ function of R. Equality of variance among *GRM5* genotypes was tested with the ‘leveneTest()’ function from the R package ‘car’ (Fox and Weisberg 2018).

Inter-class correlation coefficients (ICCs) for each variable were calculated with the training dataset using the R package ‘psych’ (Revell 2021). For all ICC, LDA, QDA and MANOVA analyses, transformed data was used when needed (Table1) and missing values were imputed using the ‘imputeMFA()’ function (‘missMDA’ R package, Josse and Husson 2016).

## Results

### Discriminant Model for *GRM5* Genotypes

Based on the mean value and the coefficient of variation (CV) of several grazing behaviours calculated from 15-days repeated measurements, 35 grazing behaviours variables were assessed as candidates for a discriminant model of the *GRM5* genotypes. Several iterations of linear discriminant analyses were run with the training dataset (*n* = 80, four individuals per genotype [*n* = 5, *i.e.*, *AB*, *AB*, *BB*, *BC*, and *CC*] and per farm [*n* = 4]). The relevance of each behavioural variable was assessed using their pooled linear discriminant scores. Furthermore, the misclassification rates obtained with each combination of variables in the corresponding discriminant models were compared (data not presented) and variables either were kept (increased accuracy) or discarded (diminished accuracy) from the model. The combination of variables was then fitted into regression models to assess their multi-collinearity with the variance inflation factor (VIF). Firstly, a quadratic discriminant model (QDM) was built with the combination of eleven grazing behaviours with highest discriminant scores and that displayed non-collinearity (see top of Table 2). Next, a model was generated with a selection of high-scored variables, which were excluded from the first model because of multi-collinearity (bottom of the Table 2).

**Table 2:** List of selected grazing personality behaviours (GP-behaviours) used in the elevation (top of the table) and exploration (bottom of the table) quadratic discriminant models (QDMs) of *GRM5* variation and their associated descriptive statistics

Grazing personality behaviours^a^	VIF^b^	LDA^c^ pooled scores	ICC2^d^ (Mean CV^e^)	MANOVA^f^ per genotype (*P-*value)	ANOVA^g^ per genotype (*P-*value)	ANOVA^g^ per *GRM5* variant (presence/absence) (*P-*value)		
						*A*	*B*	*C*
slope_mean	10.59	4.66	0.34	0.657	0.463	0.394	0.337	0.406
**rel_ele_mean**	6.16	3.89	0.26	0.272	0.120	0.241	*0.089*	0.398
rel_ele_mean_cv	3.34	3.42	(− 0.71)^e^	0.223	0.132	0.314	0.489	0.144
ele_range	5.82	3.08	0.31	0.183	**0.004**	0.102	**0.004**	**0.001**
**ele_gain**	4.23	2.98	0.45	*0.086*	*0.053*	*0.063*	*0.054*	*0.064*
rel_ele_range_cv	2.19	2.76	(− 1.06)^e^	**0.036**	*0.057*	**0.003**	0.564	0.300
ele85_farm_cv	3.94	2.61	(− 0.74)^e^	0.577	0.253	0.309	0.112	0.113
**slope15**	8.17	2.58	0.29	0.486	0.135	0.240	0.493	0.471
sp_tortuosity	3.23	1.66	0.3	0.341	0.207	*0.063*	0.695	0.488
**ele85_farm**	3.43	1.41	0.25	0.210	0.239	0.476	*0.058*	*0.058*
slope_mean_cv	2.17	0.86	(− 1.55)^e^	0.957	0.887	0.513	0.630	0.588
**dist_ho**	8.73	3.49	0.65	0.837	0.542	0.310	0.135	0.220
slope_mean	7.01	3.26	0.34	0.657	See above			
**slope_max**	5.65	3.15	0.22	0.417	0.403	0.699	0.206	*0.051*
sp_tortuosity	9.38	2.9	0.3	0.341	See above			
rel_ele_mean_cv	2.39	2.83	(− 0.71)^e^	0.223	See above			
**hr_mcp**	9.14	2.68	0.16	0.251	0.251	0.386	0.160	*0.068*
**adj_dist_ho**	2.56	2.39	0.35	**0.009**	**0.002**	**0.026**	*0.057*	**0.003**
rel_ele_range_cv	2.15	2.28	(− 1.06)^e^	**0.036**	See above			
ele85_farm_cv	2.90	1.96	(− 0.74)^e^	0.577	See above			
ele_range	3.12	1.55	0.31	0.183	See above			
slope_mean_cv	2.21	1.12	(− 1.55)^e^	0.957	See above			

*GRM5* = glutamate metabotropic receptor 5 gene exon five region with five genotypes (*i.e.*, *AB*, *AC*, *BB*, *BC* and *CC*) and three variant sequences (*i.e.*, *A*, *B* and *C*)

^a^See GP-behaviours abbreviations and details in Table 1. Bold font indicates exclusive GP-behaviour for the corresponding elevation (top of the table) or exploration (bottom of the table) discriminant model

^b^VIF = Variance inflation factor

^c^LDA pooled scores = Sum of the four absolute linear discriminant scores in the final selection of variables

^d^ICC2 = Inter-class correlation coefficient in two-way random-effects model, where cows and GPS-tracking collars were randomly allocated

^e^Mean CV = Mean of the coefficient of variation of a GP-behaviour across all cows

^f, g^MANOVA and ANOVA = *P-*values of (multivariate) analysis of variance between genotypes and variants, respectively. Variants’ presence/absence comparisons. *P* < 0.1 in italic and *P* < 0.05 in bold

The first discriminant model exclusively included variables related to elevation (elevation gain [ele_gain], the 85th quantile of elevation centred per farm [ele85_farm], relative elevation mean [rel_ele_mean]) and the 15th quantile of slope [slope15]) (Table 2). The second model included horizontal distance travelled (dist_ho), home range (hr_mcp), maximum slope (slope_max) and the adjusted horizontal distance travelled (adj_dist_ho). Both models shared seven behavioural variables: mean slope (slope_mean) and slope_mean_cv, elevation range (ele_range), CV of the relative elevation range (rel_ele_range_cv), rel_ele_mean_cv, ele85_farm_cv and movement tortuosity (sp_tortuosity) (Table 2).

Other variables with high discriminant scores, but that were not used in either model were vertical distance travelled (dist_ve), mean elevation centred per farm (ele_mean_farm), 15th quantile of elevation centred per farm (ele15_farm), 85th quantile of slope (slope85), adjusted elevation (adj_ele_mean), relative elevation range (rel_ele_range) and relative slope range (rel_slope_range). Most CV variables had low discriminant scores and were excluded from further analysis, except those included in both discriminant models (*i.e.*, CV of relative mean elevation [rel_ele_mean_cv], relative elevation range [rel_ele_range_cv], 85th quantile of elevation centred per farm [ele85_farm_cv] and mean slope [slope_mean_cv]).

The first model had VIF below 6.5 for most variables (9 out of 11), except slope15 (VIF = 8.2) and slope mean (VIF = 10.6), which were considered acceptable and without multi-collinearity (Table 2). The combination of variables selected (Table 2, top) displayed heteroscedasticity as per the Box’s Mtest (*P* < 0.001) and hence a quadratic discriminant analysis (QDA) was preferred over a linear discriminant analysis (LDA), because QDA assumes a different variance matrix for each dependable variable. This model achieved 86% accuracy to ascertain genotype (*GRM5* genotype *AB* = 69%, *AC* and *CC* = 87%, *BB* and *BC* = 94%) with the training data, which dropped to 46% when used for predicting the cow’s genotype of the testing dataset. True classifications for cows of the testing dataset were *AB* = 43%, *AC* = 50%, *BB* = 54%, *BC* = 56% and, *CC* = 31%. The *GRM5* genotype mean discriminant scores of the first model are presented at the top of Table 3.

**Table 3:** Mean discriminant scores of glutamate metabotropic receptor 5 gene (*GRM5*) genotypes for two quadratic discriminant models (QDMs)

Grazing personality behaviours^a^	*GRM5* genotype				
	*AB*	*AC*	*BB*	*BC*	*CC*
slope_mean	− 0.1500	0.3386	-0.0311	− 0.0289	− 0.1274
**rel_ele_mean**^**b**^	− 0.2191	0.4792	0.0241	− 0.1923	− 0.0918
rel_ele_mean_cv	− 0.4999	− 0.3079	0.2075	0.1304	0.4698
ele_range	− 0.1339	0.4217	− 0.4278	0.0608	0.0792
**ele_gain**	− 0.1274	0.5912	− 0.3583	− 0.0130	− 0.0925
rel_ele_range_cv	− 0.1582	− 0.0859	− 0.1979	0.5272	− 0.0852
ele85_farm_cv	0.1484	− 0.3569	0.1854	0.0072	0.0159
**slope15**	− 0.1446	0.3750	0.0031	0.0063	− 0.2398
sp_tortuosity	0.1399	0.3051	0.0378	− 0.0769	− 0.4058
**ele85_farm**	− 0.2858	0.4805	− 0.2281	− 0.0045	0.0379
slope_mean_cv	0.0668	0.0702	0.0486	− 0.0118	− 0.1737
**dist_ho**	− 0.0366	0.2287	− 0.1877	− 0.0467	0.0423
slope_mean	− 0.1512	0.3386	− 0.0311	− 0.0289	− 0.1274
**slope_max**	− 0.3518	0.2472	− 0.1590	0.1721	0.0915
sp_tortuosity	0.1399	0.3051	0.0378	− 0.0769	− 0.4058
rel_ele_mean_cv	− 0.1582	− 0.0859	− 0.1979	0.5272	− 0.0852
**hr_mcp**	− 0.1893	− 0.0499	− 0.3095	0.1124	0.4363
**adj_dist_ho**	− 0.4296	− 0.1473	− 0.3275	0.2542	0.6502
rel_ele_range_cv	− 0.4999	− 0.3079	0.2075	0.1304	0.4698
ele85_farm_cv	0.1484	− 0.3569	0.1854	0.0072	0.0159
ele_range	− 0.1339	0.4217	− 0.4278	0.0608	0.0792
slope_mean_cv	0.0668	0.0702	0.0486	− 0.0118	− 0.1737

Mean discriminant scores of QDMs per *GRM5* genotype for the corresponding grazing personality behaviours (GP-behaviours). QDMs built with scaled data (*i.e.*, centred by their mean using the R function ‘scale()’)

The top of the table lists GP-behaviours used in the ‘elevation discriminant model’. The bottom of the table list GP-behaviours of the ‘exploration discriminant model’

^a^See GP-behaviours abbreviations and details in Table 1

^b^GP-behaviours exclusive for each corresponding model are in bold

The second model had VIF values below 6 for 8 variables and no variable exceeded 10 meaning there were no multi-collinearity issues. Heteroscedasticity was detected with the Box’s M test and QDA was applied to build a discriminant model. For the training dataset, the second model achieved 87.5% accuracy, which dropped to 85% when predicting genotypes of the testing dataset. The addition (or suppression) of home range in this model did not change the model’s accuracy for the training data but increased its prediction accuracy from 81 to 85% with the testing dataset. Refer to the bottom of Table 3 for the mean discriminant scores of the second model.

### Differences Among *GRM5* Genotypes and Variants

#### Combined Behaviours (Multivariate Analysis)

**Fig. 1:**
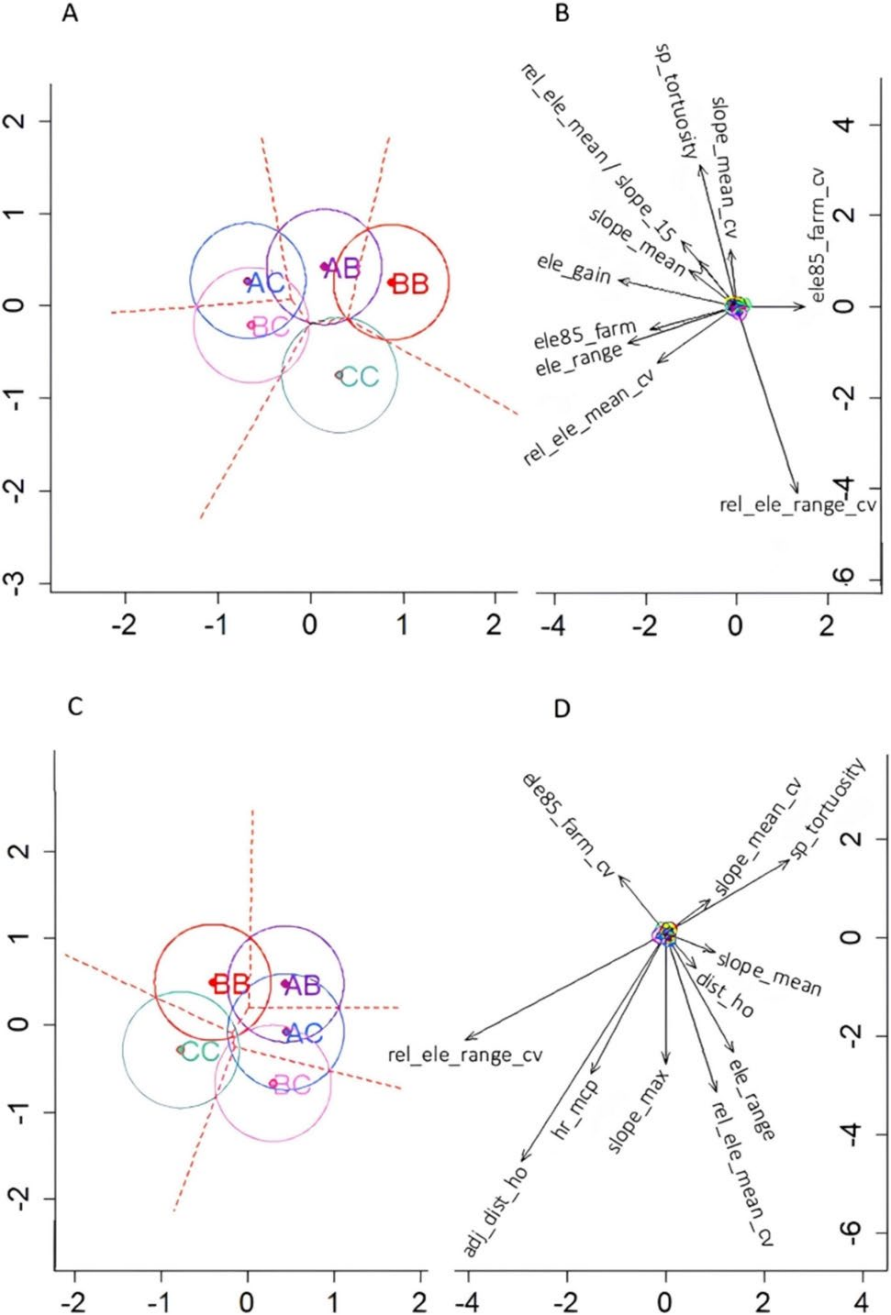
MANOVA biplots of selected grazing behaviours. Two sets of plots are obtained from the ‘elevation discriminant model’ (A and B) and the ‘exploration discriminant model’ (C and D) with axis 1 (horizontal) and axis 2 (vertical). Ellipses of confidence regions (Bonferroni method) (A and C) and selected variables used in the corresponding quadratic discriminant models (B and D) are shown. Figure coordinates were re-scaled to optimal matching

Plots of the MANOVA analyses are presented in Fig. 1A and B for the first model, and in Fig. 1C and D for the second model. Figures 1A and C reveal *GRM5* genotype ellipses for confidence level regions calculated with the Bonferroni method. Grazing behaviour variables of each respective model are presented in Fig. 1B and D.

The first two axes of MANOVA accounted for 83.0% and 81.3% of the total behavioural variation in the first and second models, respectively. For the first model, along the axis 1 (horizontal), *AC* and *BC* were paired towards the left end (negative values), *BB* occupied the opposite end, towards the right side (positive values), and *AB* and *CC* were located approximately in the centre. Genotypes *AB*, *AC* and *BB* were slightly above the origin of the axis 2 (vertical), *BC* was located slightly below zero and *CC* occupied the lowest position. Differences among genotypes on the axis 1 were mostly explained by grazing behaviours related to elevation, such as elevation gain (ele_gain), elevation range (ele_range), 85th quantile of elevation centred per farm (ele85_farm), and the coefficients of variation of the relative mean elevation (rel_ele_mean_cv) and the 85th quantile of elevation (ele85_farm_cv) (Fig. 1B). Differences along axis 2 were largely explained by the trade-off between the CV of relative elevation range (rel_ele_range_cv) and movement tortuosity (sp_tortuosity) (Fig. 1B). Relative mean elevation (rel_ele_mean) and slope-related behaviours (*i.e.*, CV of mean slope [slope_mean_cv], mean slope [slope_mean] and 15th quantile of slope [slope15]) split their contribution between axes 1 and 2.

For the second model, axis 1 revealed that genotype *CC* had the lowest values at approximately − 1, *BB* near − 0.5 and genotypes *AB*, *AC* and *BC* with similar values around 0.5. Along axis 2, *BC* occupied the lower end, *AC* and *CC* were central, and *BB* and *AB* took the upper end.

In the ellipses of both models (Fig. 1A and C), *CC* had the least overlap of confidence level region, sharing a relatively small area with *BB* and *BC*. The *BB* genotype confidence level ellipse overlaps with *AB*, *AC* and *CC* (Fig. 1A). In both graphs, there is relatively large overlap of the confidence level ellipses between *AC* and *BC*, and between *AC* and *AB*.

The MANOVA analysis of the first model resulted in a trend towards a difference for ele_gain, where the major contribution is in axis 1 and significant difference for rel_ele_range_cv corresponding to axis 2 variation (Table 2, top). For the second model, significant differences were detected for the adjusted horizontal distance (adj_dist_ho) and rel_ele_range_cv with contributions split over both MANOVA’s axes (Table 2, bottom).

#### Individual Behaviours (Univariate Analysis)

Two-way ANOVA analyses among the *GRM5* genotypes revealed differences and trends towards differences between *GRM5* genotypes for elevation range, elevation gain and adjusted horizontal distance travelled; as well as for the CV of the relative elevation range (Table 2). No differences were revealed for genotype comparisons of ele85_farm (see variant comparisons below). The two-way ANOVA revealed a farm effect for all the grazing behaviours except ele_gain and ele_range, but no farm effect was observed for ele85_farm and rel_ele_range_cv. No interactions between genotypes and farms were detected.

**Fig. 2:**
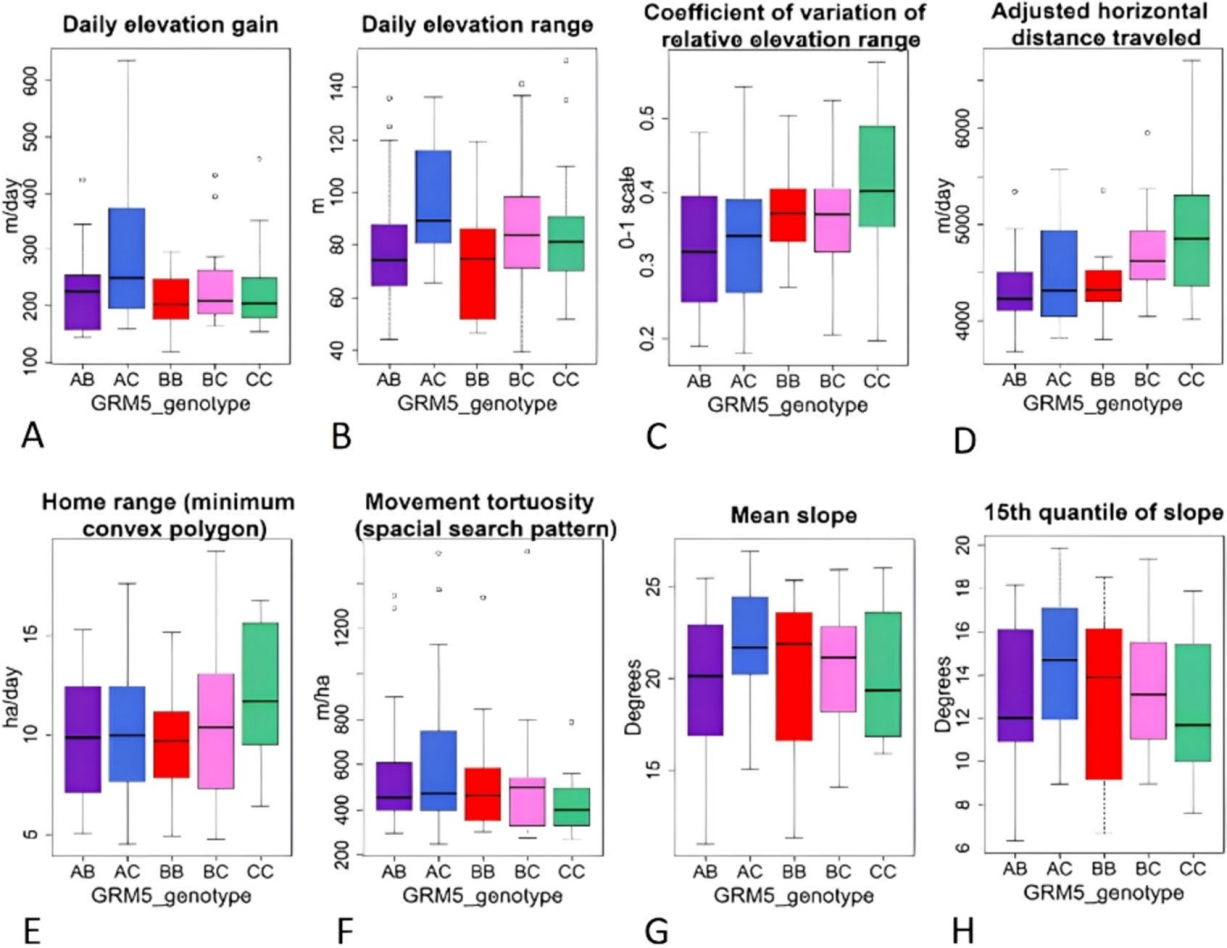
Grazing personality behaviours (A-H) box plots per genotype of the glutamate metabotropic receptor 5 gene (*GRM5*). ANOVA analyses in Table 2 indicated trends to differences (A and C) and differences (B and D). No further differences were detected. Boxes indicate the 50th (median line), 25th and 75th quantiles. The lower and upper whiskers indicate the smallest and largest values within 1.5x inter-quantile range. Empty circles display possible outliers

Figure 2 presents box plots of selected grazing behaviours per genotype produced with the original data (unscaled and untransformed), and therefore reflects the actual measured values for each genotype. Figures 2A–D correspond to behaviours with differences (ANOVA *P* < 0.1,) and Fig. 2E–H show behaviours without detected differences (ANOVA *P* > 0.1,) in the measured values.

For elevation gain (Fig. 2A) cows with genotype *AC* displayed the highest values (250 m median ele_gain), while the other genotypes had lower and approximately similar values (*AB* = 226 m, *BB* = 203 m, *BC* = 209 m, and *CC* 204 m). For ele_range  (Fig. 2B), *AC* had a median of 89 m, *AB* and *BB* displayed the lowest values of 74 m. Genotype *BC* (84 m) and *CC* (81 m) were higher, likely due to there being a few cows with extremely high scores (possible outliers shown with open circles).

The CV of the relative elevation range (rel_ele_range_cv, Fig. 2C) revealed a pattern where genotypes with the *A* variant (*AB*, *AC*) had the lowest values. Most cows with the *B* variant (*BB*, and *BC*, but not *AB*) had medium values, and the homozygous *CC* genotype cows displayed the highest CV of elevation range.

Adjusted horizontal distance travelled (adj_dist_ho) and home range (hr_mcp) had similar patterns, where genotypes *AB*, *AC* and *BB* had the lowest and similar medians (∼ 4300 m/d; ∼ 9.8 ha/d), *BC* was higher (4621 m/d; 10.4 ha/d) and *CC* had the highest median (4854 m/d; 11.7 ha/d). Similar patterns (but opposite in values) were revealed for movement tortuosity (sp_tortuosity) (Fig. 2F) where *CC* cows had the lowest tortuosity (399 m/ha), while *AB*, *AC* and *BB* had higher medium scores (453 m/ha, 472 m/ha and 462 m/ha respectively), and *BC* cattle displayed the highest tortuosity (498 m/ha).

Figure 2G, H shows the mean slope and the 15th quantile slope, respectively. In this case, cattle with the genotypes *AB* (20.1°, 12.0°) and *CC* (19.4°, 11.7°) occupied the gentlest slopes, *AC* (21.7°, 14.7°) and *BB* (21.9°, 13.9°) occupied the steepest slopes. Genotype *BC* cattle (21.2°, 13.1°) had medium slope and the 15th quantile slope values.

### Differences Among *GRM5* Variant Sequences

The ANOVA results for the presence/absence of *GRM5* variants were congruent with those results presented for the genotypes in the above section. For example, variation in ele_range was associated with variants *B* and *C*, while the three variants had trends to differences (*P* < 0.1) for ele_gain. Similarly, adj_dist_ho had differences for variants *A* and *C*, while a trend was reported for variant *B*. The variant ANOVAs also revealed differences that were not observed for the comparisons between genotypes. For example, differences and trends to differences were revealed for rel_ele_mean (variant *B*), hr_mcp (variant *C*), sp_tortuosity (variant *A*), ele85_farm (variants *B* and *C*) and slope_max (variant *C*) (Table 2).

**Fig. 3:**
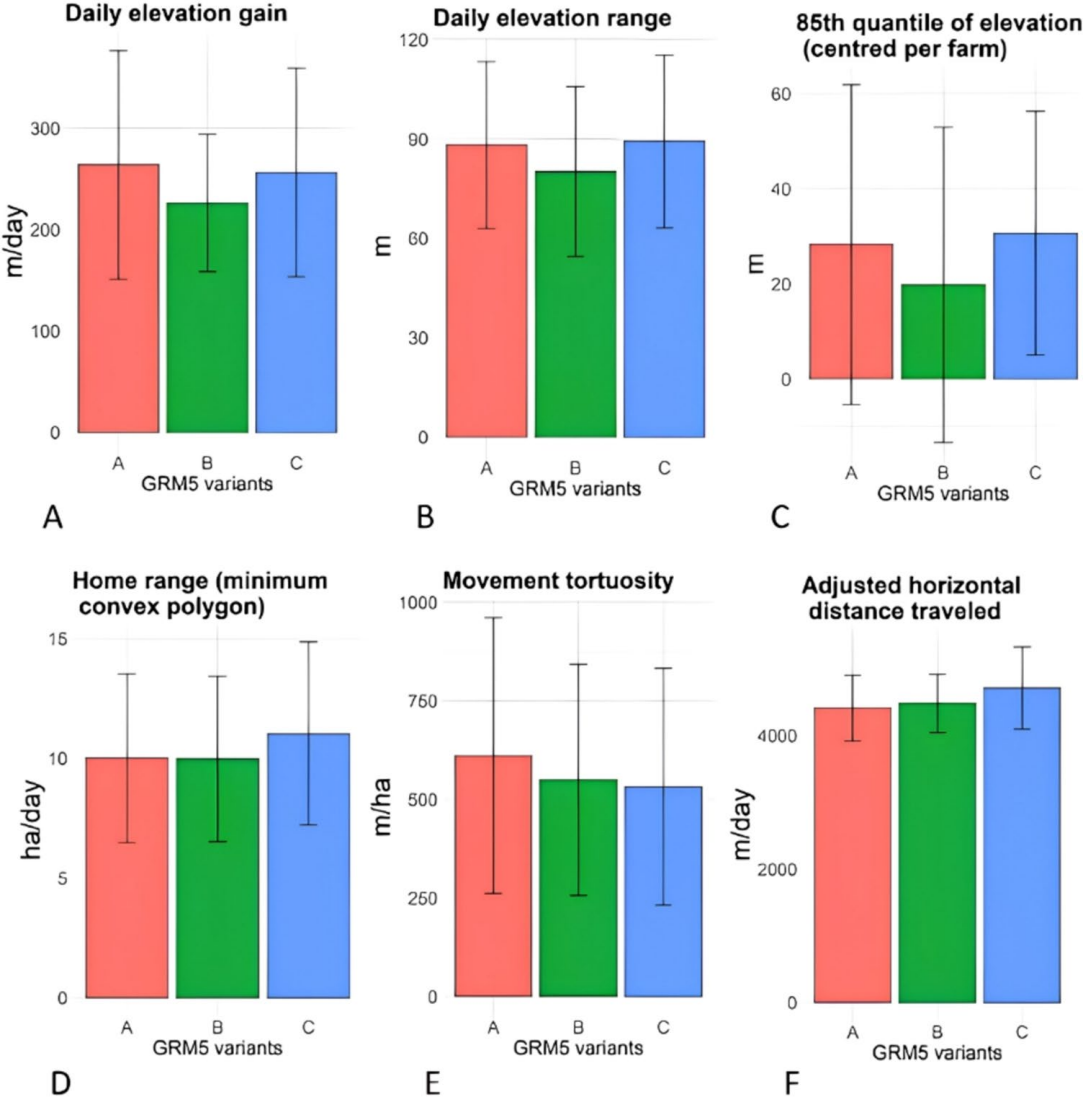
Bar plots of grazing behaviours per variant sequence of the glutamate metabotropic receptor 5 gene (*GRM5*). The error bars represent the mean values for the presence of the variant and the lower and upper whiskers indicate the standard deviation

Bar plots of grazing behaviour variables per *GRM5* variant sequence, based on the measured data, are presented in Fig. 3. For elevation-related behavioural variables such as ele_gain, ele_range and ele85_farm (Fig. 3A, B and C), variant *B* had the lowest values, while *A* and *C* displayed approximately similar and higher values. For example, ele_gain was 264 m and 256 m for variants *A* and *C* respectively, while *B* had an elevation gain of 226 m. Similarly, the elevation range of *A* and *C* was 88 m/d and was 80 m/d for *B*. These differences between *GRM5* variant sequences might be due to the high values corresponding to the *AC* genotype (Fig. 2A, B and C), rather than to the contribution of the remaining genotypes, which had similar lower values (*i.e.*, *AB* for variant *A*; and *BC*, *CC* for variant *C*).

The *C* variant cattle had the greatest home range (11 ha/d), and lower home ranges were calculated for variants *A* and *B* (10 ha/d). The movement tortuosity decreased from the highest value for variant *A* (610 m/ha) to *B* (549 m/ha), and was slightly lower again for *C* (531 m/ha).

### The Temporal Consistency of Grazing Behaviours

Inter-class correlation coefficients in two-way random effects model (ICC2) were calculated for the grazing behaviour variables of the training data using the 15-day-periods of repeated measurements. The ICC2 ranged from a minimum of 0.16 (*P* < 0.001) for relative slope range and home range to a maximum of 0.65 (*P* < 0.001) for the horizontal distance travelled (dist_ho) (Table 2). The variable dist_ho had the highest ICC2 coefficient (0.65) followed by the 15th quantile of elevation centred by farm (ele15_farm; 0.48), vertical distance travelled (dist_ve; 0.47), elevation gain (ele_gain; 0.45), and the adjusted elevation (adj_ele_mean; 0.40). Two of these grazing variables with high ICC2 values were included in the discriminant models (ele_gain in the first model and dist_ho in the second one), while the others remained unselected because they caused multi-collinearity problems.

## Discussion

The results of our *quasi-*manipulative experiment revealed a selection of seven grazing behaviours used in both discriminant models, plus four behaviours used in the first model and other four in the second one, making a total of 15 selected behaviours. Both models yielded accuracies above 86% for the training dataset, yet only one maintained similar accuracy (85%) in predicting cows’ *GRM5* genotypes when validated with the testing dataset. Our results therefore confirm linkages between cows’ *GRM5* genotypes and multiple grazing behaviours, which may result in them having distinctive grazing patterns. The *GRM5* pleiotropic effects on multiple grazing behaviours may therefore have implications for the ecological functioning of grasslands. Our analysis also highlights the advantages of setting up a *quasi-*manipulative experiment to the study of genetic linkages with the grazing behaviour of beef cattle.

### Grazing Behaviours Linked to *GRM5*

It is unsurprising that the results presented in this research are in line and supported by the ones reported by Moreno García et al. (2020), given a subset of cows from the original study were analysed here. For example, our analysis here also revealed a trade-off between home range and movement tortuosity, two grazing behaviours that were included in the discriminant models. Perhaps, the most value added with the analysis of a better-balanced dataset is in revealing possible genetic linkages with elevation- and slope-related behaviours that passed unnoticed in Moreno García’s et al. (2022) analysis, as well as with variation metrics of grazing behaviours.

In the original study from where the subset of cattle for the present study were derived, Moreno García et al. (2022) revealed associations between *GRM5* sequence variation and home range and movement tortuosity, as well as trends for association with elevation range and horizontal distance travelled. These discoveries were produced using a dataset derived from the movement of 303 mature cows randomly selected from existing commercial herds. In that experimental design, the genotypic frequency of *GRM5* was the result of artificial selection (as part of the management of the cattle for production purposes) and natural random effects occurring on those commercial farms where variant *A* was under-represented.

Four coefficients of variation (CV) of grazing behaviours had relatively high discriminant scores and were therefore included in our models, while most remaining CV metrics were disregarded due to their low discriminant scores. This highlights the importance of variation in elevation- and slope- related behaviours to differentiate grazing patterns among individual cows. Such variation in behavioural metrics has been mostly neglected (but see heart rate variation variables in Graunke et al. 2013) in prior research dealing with the grazing behaviour of beef cattle (Bailey et al. 2006; Haskell et al. 2014; Howery et al. 1996; Moreno García et al. 2022; Pierce et al. 2020). In comparison, the usefulness of behavioural variation metrics has been reflected in behaviour classification studies that include them in discriminant models applied to beef cattle (Brennan et al. 2021; Watanabe et al. 2008). Yu et al. (2021) tested the accuracy of several machine learning techniques (including linear discriminant models) to discriminate animal behaviours in several species (*i.e.*, dairy cow [*Bos taurus*], the common crane [*Grus grus*], the griffon vulture [*Gyps fulvus*], the roe deer [*Capreolus capreolus*] and the white stork [*Ciconia ciconia*]), using accelerometer-derived data. Even after applying a great reduction of variables (from 78 to 12–15 metrics), the authors built models that included variation metrics of behaviour, and that achieved relatively high discrimination accuracies.

Mean grazing behaviours used in both models included the mean slope, elevation range and movement tortuosity in agreement with the original analysis conducted by Moreno García et al. (2022). The first model also utilised elevation-related behaviours (*i.e.*, elevation gain, 85th quantile of elevation centred per farm and relative mean elevation) and therefore, it is referred to as the ‘elevation discriminant model’. The second model added exploration-related behaviours (*i.e.*, horizontal distance travelled, adjusted horizontal distance travelled and home range), so it is called the ‘exploration discriminant model’.

Previous research in animal models reported genetic associations between similar mean behaviours and the variation in *GRM5*. For example, Jew et al. (2013) found the exploratory behaviour responses of mice in a way that individuals with knock-down expression of *GRM5* in principal cortical neurons, exhibited a genetic effect of increasing total distance travelled, movement time and movement speed, and increased vertical activity in an open field experiment. Another mice experiment conducted by Wu et al. (2020), reported that the *GRM5* variation altered the total horizontal and vertical distances travelled, as well as the total time in movement of individuals undergoing an open field assay and a home cage test, respectively. In beef cattle, Bailey et al. (2015) reported associations between a quantitative trait locus containing *GRM5* and combined indexes of horizontal and vertical distances to water, slope and elevation measured in free-ranging cows in steep and rugged grasslands in USA. The original study reported by Moreno García et al. (2022) also revealed associations and trends towards association with some of the grazing behaviours selected for the discriminant models here (*i.e.*, home range and movement tortuosity and horizontal distance travelled and elevation range, respectively). However, a better-balanced dataset chosen for the present analysis, allowed the building of an elevation and an exploration discriminant models that revealed possible *GRM5* linkages with elevation-, exploration- and slope-related behaviours, as well as with metrics of behaviour variation.

### Discriminant Models of *GRM5* Genotypes

#### Models’ Variables

The elevation and exploration discriminant models included selected metrics of variation (*i.e.*, CV of relative mean elevation and relative elevation range, CV of 85th quantile of elevation centred per farm, and CV of mean slope). Such variation variables have not been previously tested for genetic association nor considered as distinctive features among individual cattle.

In the elevation discriminant model, the behavioural variation among *GRM5* genotypes was primarily driven by a first axis of elevation-related behaviours (*i.e.*, ele_gain, ele_range, ele85_farm, rel_ele_mean_cv, and ele85_farm_cv) and a second axis characterized by the movement tortuosity (sp_tortuosity) and the variability in elevation range per cow relative to the elevation range of the herd (rel_ele_range_cv) (Fig. 1B). Slope-related metrics (*i.e.*, mean slope and its 15th quantile) contributed to the variation in both axes, with a larger effect on the second one (Fig. 1B). Bailey et al. (2015) tested genotype-to-phenotype associations between the whole genome (*i.e.*, ∼ 778 thousand SNPs single nucleotide polymorphisms) using an Illumina BovineHD BeadChip to analyse mature beef cows, and indexes of terrain use derived from cow GPS relocation data (25–112 days recorded, and 96–288 GPS fixes per day). The cattle in the study of Bailey et al. (2015) included a relatively small group (*n* = 87) of lactating and non-lactating cows of various breeds (*i.e.*, Angus, Angus x Hereford cross, Brangus, Limousin, and Simmental-crosses) under free-range grazing on five ranches in Arizona, Montana and New Mexico, USA. The terrain was purposely chosen to be diverse, including having rolling and mountainous areas with gentle to moderate slopes, as well as undulating plains. The analysis of Bailey et al. (2015) revealed associations between genetic variation of QTL overlapping* GRM5* and a ranking index that combined slope and elevation (called the ‘rough index’). Their results support the findings described here, where axis 1 of the elevation discriminant model was mainly explained by elevation and slope behaviours (Fig. 1B).

On the other hand, the axis of highest behavioural variation in the exploration discriminant model (axis 1) was primarily explained by the opposite effects of rel_ele_range_cv and sp_tortuosity (Fig. 1D), which resembles axis 2 of the elevation discriminant model (Fig. 1B). Adjusted horizontal distance travelled (adj_dist_ho) and home range (hr_mcp) had large, but split contributions to axes 1 and 2 of the exploration model. These two behaviours along with the CV of relative elevation mean (rel_ele_mean_cv), maximum slope (slope_max) and elevation range (ele_range), were the main drivers of variation in axis 2. Another index with reported associations to *GRM5* genotypes in the Bailey’s et al. (2015) study combined elevation, slope, and distance to water (*i.e.*, ‘rolling index’). This could not be evaluated in the current study, because the paddocks commonly had several sources of water, rendering such calculation inappropriate. However, other proxy behaviours of grazing exploration, such as the horizontal distances travelled, adjusted horizontal distance travelled and home range, were major components of the exploration discriminant model (Fig. 1D). The importance of elevation-, slope- and exploration-related behaviours that emerged from discriminant models is consistent with the genetic associations reported by Bailey et al. (2015) and they are also consistent with associations reported for *GRM5* mice models [*e.g.*, associations to horizontal and vertical distances (Wu et al. 2020), locomotor reactivity (Jew et al. 2013; Wu et al. 2020) and trajectory patterns (Bakker and Oostr 2003)].

The large and opposing effects of sp_tortuosity and rel_ele_range_cv were observed in both models (Fig. 1B axis 1 and Fig. 1D axis 2), suggesting a trade-off between these two behaviours. Furthermore, axis 2 of the exploration model showed opposite effects of sp_tortuosity towards the positive side, and hr_mcp and adj_dist_ho towards the negative end of the axis. This agrees with the trade-off between movement tortuosity and home range reported in Moreno García et al. (2022), and further supported by Browning et al. (2018) and Pauler et al. (2020).

Among the high-scoring grazing behaviours (that were excluded from both discriminant models) were vertical distance travelled, 85th quantile of slope, mean and 15th quantile of elevation; which although relevant, might be redundant with behaviours included in the models. Some of the so-called relative behaviours (*i.e.*, metrics comparing behaviours of individual cows *versus* behaviours of the herd) were also among the high-scored grazing behaviours not included in discriminant models. For example, adjusted daily elevation mean, relative elevation range and relative slope range. Most CV behaviours scored low in the initial discriminant analysis and might only have reduced importance in discriminating between the *GRM5* genotypes.

#### Models’ Accuracy

In a paired comparison, Watanabe et al. (2008) tracked the behaviour of a Holstein cow and a Japanese black cow under barn and pasture grazing conditions (respectively) using three-axis accelerometers deployed under the animal’s jaw. They calculated the mean, variance, and inverted CV of the acceleration of under-jaw movement per min by aggregation of 1-s frequency measurements and for each of the three accelerometer axes. Metrics were also computed for the resultant axis, making a total of twelve variables. The authors tested several combinations of the twelve variables to build QDMs of cows’ activities (*i.e.*, eating, ruminating, and resting), which were determined by observing time-synchronized video recordings. The QDMs achieved ∼ 95% accuracy with the training dataset (the models’ accuracy were not evaluated with other cows) when they included eight means and inverse CVs variables or all twelve variables.

In a similar fashion to the Watanabe et al. (2008) findings, the discriminant models described here included aggregated metrics of means and of repeated measurements variation (*i.e.*, inverse CV in Watanabe’s et al. and CV here), but our models achieved ∼ 86% accuracy, which is roughly 10% less than the Watanabe’s et al. (2008) discriminant model. The lower accuracy of the discriminant models created here might be attributable to several reasons. First, our model attempted to discriminate five *GRM5* genotypes measured in several cows (*n* = 80) and therefore involves within-individual and among-individual variation, while Watanabe’s model discriminated among fewer groups (*i.e.*, three behaviours) measured in two cows, which therefore accounted mostly for within-individual variation, which is hierarchically smaller than the among-individual variation (Westneat et al. 2015). Adding among-individual variation may create a larger overlap between the discriminating categories, likely causing a reduction in a model’s accuracy. Lower model accuracy may also occur due to a relatively larger plasticity of *GRM5-*controlled grazing behaviours derived from GPS and satellite image data, when compared to accelerometer-derived metrics. For example, in Watanabe’s model, the absolute values of CV variables ranged between 7.67 and 0.34 (calculated based on published data), while in the models presented here, the CV of the 85th quantile of elevation centred per farm (ele85_farm_cv) ranged between 83.74 and 0.04, reflecting a much larger variation in support of the above-mentioned argument. However, the remaining three CV variables used in the elevation and exploration models had smaller variations than in Watanabe’s model (ranging between 0.75 and 0.04). The exclusion of ele85_farm_cv from the elevation and exploration models causes a reduction in accuracy of 1.25% and 11.25%, respectively. Since these models are based on grazing behaviours derived from GPS and satellite data (instead of accelerometer-derived metrics), one could question whether there might be more suitable behavioural metrics to discriminate *GRM5* genotypes. Other factors that might cause decreased model accuracy are the level of control imposed by *GRM5* over grazing behaviours (*i.e.*, to what extent does *GRM5* variation determine grazing behaviours?) and the interaction of *GRM5* with other gene(s) that might affect grazing behaviours (are there polygenic and/or pleiotropic effects on *GRM5*-controlled grazing behaviours?).

Brennan et al. (2021) reported an averaged accuracy of 85% over 3 years (ranging from 80–92%) for QDMs discriminating between grazing and non-grazing behaviours in free ranging yearling steers. Again, these models included a mix of variables with mean, maximum, minimum, and standard error metrics, but in this case the data were derived from accelerometer and GPS devices. They analysed data of free-ranging steers grazing native grasslands of South Dakota (USA) over ∼ 90-day periods and in three consecutive summers (2016–2018). Brennan’s QDMs accuracy was like the ones reported here, yet lower than other publications. The authors hypothesized that the longer tracking periods and the larger paddock size (between 51–74 ha) of their experiment, compared to other experiments, could explain such underperformance.

The elevation and exploration models herein, performed similarly to those reported by Brennan et al. (2021) (∼ 86% vs. 85% accuracy), but had a 10% lower accuracy than Watanabe’s et al. (2008) model. The latter model was built with data collected in much shorter periods (two to four sessions of 3–4 h) and this might explain its better performance, as was argued by Brennan et al. (2021). This argument is further supported by Dochtermann et al. (2015), who reported a decrease of 0.52 mean heritability to a 0.14 of behavioural variation, because the latter metric accounted for the effect of temporal variation. Despite that, our 15-days data aggregation is a lot shorter than the 90-days used in Brennan’s et al. (2021) model, yet only a 1% improvement in accuracy was achieved. In this regard, we suspect that the reason for a decreased accuracy of discriminant models using long periods of behavioural measurements, such as the ones presented here, might be because of an increased variation over longer time periods. The increased temporal behavioural variation is reflected here with the low ICCs achieved for most behaviours in our experiment. For example, most grazing behaviours rated ICCs below 0.35. Such low ICC values are indicative of ‘poor’ consistency (Koo and Li 2016); although this interpretation depends on the measurement under evaluation. Our ICC scores would suggest a low temporal consistency in selected grazing behaviours.

Another factor that one could have expected resulting in an increased model accuracy of our models, compared to the reported by Brennan’s et al. (2021), is the smaller paddocks size we had in our study (average 34.5 ha vs. 51–74 ha). However, we argue that paddock size combined with other features (*e.g.*, spatial vegetation and abiotic heterogeneity, paddock shape and distribution of water and shade) that might interfere with the grazing behaviour of cattle (von Müller et al. 2017; Sevi et al. 2001), and ultimately affect the accuracy of the discriminant models.

Another aspect to consider is the number of categories the model discriminates. In a simulation experiment, El-habil and El-Jazzar (2014) reported a reduction of ∼ 5% in the accuracy of a linear discriminant analysis, when the number of categories increased from 2 to 5. Ladds et al. (2017) reported an average reduction of 11.5% accuracy of four different machine learning algorithms (*i.e.*, random forests, gradient boosting machine, logistic regression, and super learner) when increasing from four categories (*i.e.*, foraging, grooming, resting, travelling) to six (feeding and thrashing added) in classification models of seal behaviours recorded with accelerometers. This might well explain the ∼ 10% lower accuracy of our models discriminating five categories compared with Watanabe’s et al. (2008) model that discriminated among three categories (*i.e.*, eating, ruminating, and resting). On the other hand, it seems remarkable that our models achieved similar accuracy to Brennan’s et al. (2021) model discriminating between two-activity statuses (*i.e.*, grazing *versus* no grazing).

Finally, we also wanted to point out the loss of accuracy of our elevation model that dropped from 85 to 46% when predicting genotypes of cows from the testing dataset. This loss of accuracy might question the feasibility of predicting *GRM5* genotypes with an elevation model, and suggesting strongly the need of further validation with other cattle. Nonetheless, the differences among *GRM5* genotypes reported for individual behaviours related to elevation (for example, elevation gain and range in Fig. 2) highlight the validity of the elevation model to discriminate cows with various genotypes. On the other hand, the exploration discriminant model that included three grazing behaviours associated with *GRM5* genotypes (*i.e.*, home range, movement tortuosity and elevation range, Moreno García et al. 2022), yielded promising results on the ability to discriminate and predict cow genotype.

### Grazing Patterns and Grassland Ecology as Affected by *GRM5* Genotypes

The two-way ANOVA (Table 2) revealed differences and trends to differences between *GRM5* genotypes and variant sequences (presence/absence models) for grazing behaviours such as ele_gain, ele_range, rel_ele_range_cv and ele85_farm (only in variants *B* and *C*) (see Table 2 and Figs. 2 and 3). In contrast, ANOVA analyses failed to reveal differences for home range (data not shown) and sp_tortuosity. However, Moreno García et al. (2022) reported differences between *GRM5* genotypes, where 4–5 years of age cows with *BB* genotype showed the smallest home range and the largest movement tortuosity and, on the opposite, *AB*/*AC* genotypes display among the largest home ranges and the least tortuous movements. While the mensurative analysis in Moreno García et al. (2022) revealed differences for these behaviours, the manipulative analysis did not. However, it highlighted the importance of both behaviours to differentiate among* GRM5* genotypes.

Altogether, these findings suggest that cows with different *GRM5* genotypes differ in their exploration patterns within steep and rugged terrain, with potential effects on the ecological functioning of grasslands. For example, at the individual level, *AB*/*AC* cows would display multiple correlated grazing behaviours, tending to graze larger areas and walking straighter trajectories, while having larger elevation gains and elevation ranges, when compared to *BB* cows. At the collective level, groups of cows with different proportions of genotypes displaying contrasting grazing behaviours (*e.g.*, 20:70:10 vs 70:10:20 of *GRM5* genotypes *BB*:*AC*:*AB*) may explore and utilize steep and rugged grasslands very differently as a group. Vegetation patches may therefore be affected differently by contrasting grazing intensities, resulting in higher or lower sward heights, and by contrasting grazing frequency dictating the resting/regrowth period of the vegetation (Vallentine 2001a; b). Such differences in grazing regime (*i.e.*, intensity + frequency) shapes the functioning strategy of plant communities (*e.g.*, speed of regrowth, photosynthetic capacity, speed of nutrient cycling; see He et al. 2021; Moreno García et al. 2014; Zheng et al. 2015) by increasing the survival rate and the successful establishment of certain plant species to the detriment of functionally contrasting species and by triggering physiological responses of vegetation that converge into plant communities with defined ecological functions.

Ultimately, if cows’ grazing behaviours as linked to their genotype can lead to shifts in plant community composition and function, the manipulation of *GRM5* genotype proportion in beef cattle herds may indirectly determine the health and the sustainability of pastoral livestock systems (Tainto 1999). For example, building cattle herds for grazing larger areas with straighter grazing path trajectories over greater elevation gain and range would be beneficial for the conservation of sensitive riparian grasslands. Or the opposite: cattle herds that graze smaller areas over reduced elevation gain and range, may be useful for the conservation of tussock grasslands in high country grasslands. The behavioural genetics of grazing may be a cost-effective tool to manipulate the utilisation and functioning of grasslands and, in so doing be useful in tackling range management challenges and conservation objectives.

### *GRM5* Pleiotropic Effects

The genetic linkages between one specific gene as investigated here using PCR-SSCP, a fine resolution high-specificity technique, and multiple grazing behaviours, suggest pleiotropic effects of *GRM5* over the grazing patterns of beef cattle.

It could also be that there are other gene(s) involved in controlling multiple behaviours that remain undetected. For example, Bailey et al. (2015) used broader resolution genetic markers to identify associations between quantitative trait loci regions and indices of terrain use that aggregate grazing behaviours, such as mean slope, mean elevation, and distance travelled to drinking points. The authors derived a list of putative genes for grazing behaviours that were likely contained within the identified QTL regions, such as the succinate dehydrogenase complex assembly factor 3 gene (*SDHAF3*, but referred to as *ACN9*), the mastermind like transcriptional coactivator 3 gene (*MAML3*), the RUN and SH3 domain containing 2 gene (*RUSC2*) and *GRM5*. For the latter, grazing behaviour associations have been reported (Moreno García et al. 2022), but no further information has been revealed for the other genes.

Another putative grazing gene in cattle is the leptin gene (*Lep*). Chilliard et al. (2005) described the regulatory mechanisms of *Lep* expression and its effects on feed intake, feeding efficiency, growth, and fertility- and immunity-related traits in cattle. The mouse leptin knock-out model (Medina-Gomez et al. 2007) reported decreased exploration activity and increased water consumption in mice with the *lep* ablation genotype. Since *lep* ablated mice also displayed feeding and growth responses (*i.e.*, storing large amounts of body fat and becoming obese), it might be expected that the bovine leptin gene may control grazing behaviours and the growth characteristics of cattle, denoting its pleiotropic effects as reported elsewhere (Fiett 2005).

More functional studies on what the *GRM5*-derived protein does are needed to increase our understanding of the underlying mechanisms that link *GRM5* with grazing patterns and, in so doing, confirm pleiotropy.

### A Quasi-Manipulative Experiment

The original study conducted by Moreno García et al. (2022) analysed grazing behaviours of cows with the given proportion of *GRM5* genotypes by the experimental conditions in a mensurative experiment (*sensu* Hurlbert 1984). Alternatively,* quasi*-manipulative experiments that ensure sampling interspersion may increase the power of an experiment to detect treatment effects (Hurlber 1984). We applied a randomized under-sampling of the most numeric classes as proposed by Haixiang et al. (2017) and built a dataset that equally represented the five *GRM5* genotypes and the four farms. The quasi-manipulative experimental design allowed the use of *GRM5* genotype as a ‘treatment’ and farm as a covariate. Our discriminant analysis approach suggests *GRM5* genetic linkages with grazing behaviours that another mensurative study was unable to detect (Pierce et al. 2020) and that even passed unnoticed when using the full dataset of the original experiment (Moreno García et al. 2022). On the other hand, there is evidence in the literature that supports our findings in other animal models (Bakker and Oostr 2003; Jew et al. 2013; Wu et al. 2020) and in a small sample size experiment with cattle (Bailey et al. 2015). What-is-more, future research with larger-scale experiments (*e.g.*, 300 to 600 individuals) and balanced representation of *GRM5* genotypes, may also improve the statistical ability to identify *GRM5*-controlled grazing behaviours.

The comparison of results between the two analytical approaches could lead to the belief that the analysis with a *quasi*-manipulative experiment was somehow better than the original mensurative approach. However, each analysis has its own singular advantage (*i.e.*, a larger sample size in Moreno García et al. 2022, and an even representation of *GRM5* genotypes in the present work) and it is notable that such different analyses, deliver similar results. Furthermore, the discriminant approach with a balanced yet much smaller dataset highlights the possibility of hidden associations that have not been detected because of the limitations of the mensurative experiment.

Future research with manipulative experiments could target predicting the *GRM5 *genotype of cows based on grazing behaviours and then genotyping to ascertain whether the predictions were correct. The use of data and new metrics derived from GPS, accelerometers (Brennan et al. 2021; Watanabe et al. 2008) and gyroscopes (Kleanthous et al. 2019) as well as the annotation with external data sources (*e.g.*, satellite- or drone- captured data) might then assist to build more robust discriminant models with higher classification accuracy.

## Conclusions

We used discriminant analyses to select combinations of key grazing personality behaviours (GP-behaviours) that discriminated specific genotypes of *GRM5*, a potential ‘grazing gene’. One quadratic discriminant model, built with eleven key GP-behaviours related to elevation, slope and exploration, correctly predicted the specific genotype of more than 85% of the free-grazing cows investigated in steep and rugged terrain in New Zealand. These results highlight the importance of behavioural genetics, animal personality and repeated measurement data to detect differences between individual herbivores grazing in steep and rugged terrain. The design of experiments with balanced genotypic variation might be a scientific alternative to using more extensive experimental setups with the advantage of having better control over variables (measured or otherwise) that potentially affect behaviour.

## Data Availability

The GPS data that support the findings of this study were deposited in https://www.movebank.org with the identifiers ID 1321429570 and ID 1321461925 and available from the corresponding author on reasonable request.
